# CAR-T Cells Hit the Tumor Microenvironment: Strategies to Overcome Tumor Escape

**DOI:** 10.3389/fimmu.2020.01109

**Published:** 2020-06-17

**Authors:** Alba Rodriguez-Garcia, Asis Palazon, Estela Noguera-Ortega, Daniel J. Powell, Sonia Guedan

**Affiliations:** ^1^Center for Cellular Immunotherapies, Perelman School of Medicine, University of Pennsylvania, Philadelphia, PA, United States; ^2^Cancer Immunology and Immunotherapy Laboratory, Ikerbasque Basque Foundation for Science, CIC bioGUNE, Basque Research and Technology Alliance (BRTA), Derio, Spain; ^3^Department of Hematology and Oncology, Institut d'Investigacions Biomèdiques August Pi i Sunyer (IDIBAPS), Hospital Clinic, Barcelona, Spain

**Keywords:** chimeric antigen receptors (CAR), solid tumors, immunotherapy, immunosuppressive tumor microenvironment, adoptive cell transfer (ACT), inhibitory receptors

## Abstract

Chimeric antigen receptor (CAR) T cell therapies have demonstrated remarkable efficacy for the treatment of hematological malignancies. However, in patients with solid tumors, objective responses to CAR-T cell therapy remain sporadic and transient. A major obstacle for CAR-T cells is the intrinsic ability of tumors to evade immune responses. Advanced solid tumors are largely composed of desmoplastic stroma and immunosuppressive modulators, and characterized by aberrant cell proliferation and vascularization, resulting in hypoxia and altered nutrient availability. To mount a curative response after infusion, CAR-T cells must infiltrate the tumor, recognize their cognate antigen and perform their effector function in this hostile tumor microenvironment, to then differentiate and persist as memory T cells that confer long-term protection. Fortunately, recent advances in synthetic biology provide a wide set of tools to genetically modify CAR-T cells to overcome some of these obstacles. In this review, we provide a comprehensive overview of the key tumor intrinsic mechanisms that prevent an effective CAR-T cell antitumor response and we discuss the most promising strategies to prevent tumor escape to CAR-T cell therapy.

## Introduction

T cells that are genetically modified to express chimeric antigen receptors (CAR-T) constitute a potent new cancer therapy with curative potential (1, 2). CAR-T cell therapy has produced impressive response rates in patients with certain B-cell malignancies, resulting in the recent approval of two CAR-T cell products targeting CD19 (3, 4). Numerous CAR-T cell therapies targeting a variety of antigens are under clinical investigation, with anti-BCMA CAR-T cells showing very promising results for the treatment of multiple myeloma (5). Despite the impressive responses in patients with hematologic malignancies, early clinical trials using CAR-T cells in patients with solid tumors have reported limited antitumor activity, with objective responses observed only in a minority of patients (6–8).

The potential of T cells to induce complete responses in patients with solid tumors has been demonstrated by the success of immune checkpoint therapy (9). Also, objective responses to adoptive T cell therapy with tumor infiltrating lymphocytes (TILs) and T cells that are genetically engineered to express a transgenic T cell receptor (TCR) have been reported in patients with melanoma, sarcoma, cholangiocarcinoma, and breast cancer (10). While only a proportion of patients exhibit long term, durable responses, these results suggest that T cells have the potential to eliminate solid tumors under adequate conditions. However, to date only anecdotes of CAR-T cell mediated response have been reported (6, 8). Understanding the mechanisms that limit CAR-T cell efficacy in solid tumors is essential to design the next-generation of CAR-T cell therapies with increased therapeutic index.

Some of the key factors limiting the applicability of CAR-T cells for the treatment of solid tumors include: the lack of truly tumor-specific target antigens (11); tumor heterogeneity and plasticity that can lead to tumor escape due to loss of antigen expression (12); T cell dysfunction driven by CAR-mediated tonic signaling (13–15) or chronic antigen exposure (16); and the immunosuppressive tumor microenvironment (TME) (17). In this review, we summarize the key challenges that CAR-T cell encounter in the TME, with a particular emphasis on tumor intrinsic factors, such us hypoxia, extracellular matrix (ECM) and stromal and immune cells. We also discuss some of the efforts that are underway to overcome these challenges and expand the therapeutic window of CAR-T cells for the treatment of solid tumors (Table 1).

**Table 1:** Main challenges for CAR-T cell therapy in solid tumors and emerging strategies to address them.

**Factors harnessing CAR-T cell therapy efficacy in solid tumors**	**CAR-T cell-based approaches proposed to overcome limitations**
**TUMOR PENETRATION**	
**Endothelial barriers**	
• Tumor vasculature	• Disrupt tumor vasculature with CAR-T cells (18–22)
**T cell exclusion from tumors**	
• Extracellular matrix (ECM) • Cancer-associated fibroblasts (CAFs)	• Express matrix-degrading enzymes (23, 24) • Target CAFs with CAR-T cells (25–34)
**TUMOR MICROENVIRONMENT (TME)**	
**Hypoxic tumor conditions**	• Chose appropriate costimulatory domains (35–37) • Restrict CAR expression to hypoxic conditions (38) • Target antigens upregulated in hypoxic conditions (39)
**Immunosuppressive immune cells**	
• Regulatory T cells (T_regs_) • Tumor-associated macrophages (TAMs) • Myeloid-derived suppressor cells (MDSCs)	• Combine CAR-T cells with antibodies that reduce T_reg_ frequencies (40–43) • Target T_regs_ with CAR-T cells (44) • Use lymphodepleting regimens to eliminate T_regs_ (45) • Reduce IL-2 availability for T_regs_ by: ° Choosing appropriate costimulatory domains (46–51) ° Mutating costimulatory domains (52) ° Using alternative cytokines to support engineered CAR-T cells (49, 53, 54) • Target TAMs with CAR-T cells (55–57) • Reeducate TAMs toward antitumor phenotype (58–64) • Combine CAR-T cells with agents that reduce MDSC content (43, 65–71) • Target MDSCs with CAR-engineered T/NK cells (72, 73)
**Immunosuppressive soluble factors**	
• TGF-β, IL-4, IDO	• Confer resistance to immunosuppressive factors by engineering CAR-T cells to express: ° Dominant-negative receptors (74) ° Switch receptors (75, 76) ° Disrupt inhibitory cytokine receptors by genome editing (77) • Engineer CAR-T cells to release support cytokines (78–84) • Combine CAR-T cells with inhibitors (85)
**IMMUNE EVASION AND SUPPRESSION**	
**Expression of inhibitory receptors and ligands by tumor and/or stromal cells**	
• PD-1/PD-L1, CTLA-4, LAG-3, TIM-3, TIGIT	• Combine CAR-T cells with immune checkpoint blockade antibodies (71, 86–90) • Combine CAR-T cells with oncolytic viruses releasing immune checkpoint inhibitors (91) • Engineer CAR-T cells to express: ° Blocking antibodies (92, 93) ° Dominant negative receptors (86) ° Switch receptors (94) • Disrupt T cell inhibitory receptors by genome editing (95–100)

## Physical Barriers

### Hypoxia

Defined as a shortage in oxygen availability, hypoxia is a prominent feature of solid tumors that results from an aberrant vascularization and rapidly proliferating tumor cells. Tumor hypoxia has been correlated with poor patient prognosis (101), resistance to neoadjuvant therapy (102, 103), and metastatic success (104). Importantly, reduced oxygenation can also influence antitumor immune responses (105).

Cellular adaptations to oxygen levels are governed by the hypoxia pathway and mediated by hypoxia-inducible factors (HIF). When oxygen is available, prolyl hydroxylase domain proteins (PHDs) are active and hydroxylate HIF, leading to HIF ubiquitination by Von-Hippel Lindau (VHL), and HIF degradation in the proteasome. When oxygen levels drop, hydroxylases become inactive leading to HIF stabilization and translocation to the nucleus, where it forms a transcriptional complex that directly binds to specific regions, termed hypoxia response elements (HREs). HREs are present in the promoters of several genes that encode for important proteins that mediate the cellular adaptation to hypoxia, such as glycolytic enzymes and the vascular endothelial growth factor-A (VEGF-A) (106). This family of transcription factors is mainly comprised of two isoforms: HIF-1α and HIF-2α (107), with HIF-1α being the main isoform expressed by activated T cells (108). HIF-1 accumulation in T cells promotes antitumor immunity in mouse models of solid tumors and metastases (109, 110).

After activation, T cells increase glucose uptake and glycolytic rate to support proliferation and the acquisition of effector functions (111). This process is supported by HIF stabilization after TCR engagement and augmented under hypoxia. A consequence of the T cell adaptation to hypoxia is metabolic rewiring, a process in which the reduced rate of oxidative phosphorylation (OXPHOS) is compensated by enhanced glycolysis. Competition for nutrients, persistent antigenic stimulation and immunosuppressive networks in the TME can lead to T cell exhaustion (112). Another consequence of metabolic adaptation in T cells is the accumulation of metabolites that impact epigenetic landscapes that influence the fate and function of T cells (113). One example is the increased production of the oncometabolite 2-hydroxyglutarate (2-HG) by hypoxic T cells. 2-HG inhibits 2-oxoglutarate-dependent epigenetic enzymes (114) resulting in the modulation of the T-cell terminal differentiation and favoring a central memory phenotype (115). Certain histone demethylases, such as KDM6A and KDM5A, can also be directly inhibited by a shortage of oxygen in a HIF- and 2-HG independent manner, leading to the control of gene expression and cell fate (116, 117).

**Figure 1:**
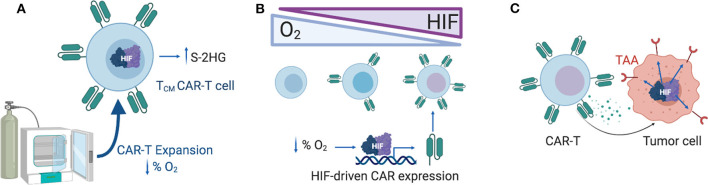
Exploiting the hypoxia response pathway for CAR-T therapy. **(A)** Expanding CAR-T cells *ex vivo* under reduced oxygen concentrations (1–5% O_2_) might support the enrichment of memory-like T cells, a process mediated by S-2HG. **(B)** CAR expression can be gradually modulated by increasing levels of HIF-1α in T cells, generating a hypoxia-responsive CAR-T with increased CAR expression in hypoxic tumors and reduced CAR expression in the periphery. **(C)** Selection of TAAs that are upregulated under hypoxic conditions in solid tumors might limit off-tumor CAR-T cell activity. HIF-1α, Hypoxia-inducible factor 1 alpha; S-2HG, S-2-hydroxyglutarate; TAA, tumor associated antigen.

The level of oxygenation impacts several aspects of CAR-T therapies (Figure 1). *In vitro*, hypoxia decreases the expansion capacity of CAR-T cells, blocking their differentiation into effector memory cells, and enriching the cultures with T cells with a central memory cell phenotype (118). Culturing and expanding CAR-T cells under controlled physiological oxygen concentrations might be an approach for enriching the cultures with memory-like T cells, which are known to have better persistence and efficacy than terminally differentiated effector T cells (119).

After infusion, CAR-T cells must infiltrate solid tumors and carry out their cytotoxic activities. How hypoxia influences these processes remains largely unexplored. Recent development of *in vitro* tools will support the study of CAR-T function in relevant oxygenation conditions (120). In this context, the use of organoids and 3D tumor models (121–123) will support the preclinical development of CAR-T cells for the treatment of solid tumors.

The hypoxia pathway offers several opportunities for the design of CAR-T cells (Figure 1). The choice of the optimal costimulatory domains in the CAR might be influenced by oxygen availability in the TME, given that the metabolic consequences of signaling downstream of CD28 and 4-1BB are different (35–37). Another attractive approach is the design of CARs that are active in the TME, but inactive in better oxygenated environments in an attempt to reduce off-site toxicities. Novel strategies to confine CAR expression to the TME consists of introducing HRE regions on the promoter of the construct, or fusing HIF domains to the intracellular domain of the CAR to promote the hydroxylation and degradation of the CAR when oxygen is available (38). Both approaches rely on the endogenous T cell oxygen-sensing machinery to control the expression of the CAR. Alternatively, CAR-T cell activity can also be targeted to antigens that are known to be upregulated under hypoxic conditions in solid tumors, such as carbonic anhydrase IX (39).

Hypoxia also promotes immunosuppressive pathways in the TME that offer combinatorial therapeutic strategies with CAR-T cell approaches. Hypoxia and HIF promote the expression of program death ligand 1 (PD-L1) (86, 124) and adenosine levels (125, 126), as well as the recruitment of regulatory T (T_reg_) cells in the TME (127), all of which are known to inhibit T cell responses.

### Extracellular Matrix

The ECM is an integral constituent of the tumor stroma composed of different macromolecules including fibrous proteins, glycosaminoglycans, and proteoglycans. The ECM is produced by tumor cells themselves as well as by cancer-associated fibroblasts (CAFs) and play an important role in cancer progression. Increased deposition of collagen or hyaluronan, constituents of the ECM, in tumors correlate with poor prognosis in different cancer types (128–131).

In addition, the ECM represents a physical barrier to various anticancer therapies, preventing their penetration and infiltration of tumors. Agents such as collagenase or hyaluronidase can degrade distinct components of the ECM and improve antitumor efficacy of diverse cancer therapies, including chemotherapy, oncolytic viruses, monoclonal antibodies, or checkpoint blockade (132–142).

While the role of ECM in resistance to adoptive T cell transfer therapies remains underexplored, some studies demonstrate that peritumoral ECM collagen fibers limit T cell access to tumors, and indeed, tumors with high-collagen density present lower levels of infiltrating T cells (142, 143). Here, the use of the matrix-degrading agents that facilitate T cell infiltration of tumors provides a rationale for matrix degradation as a means to improve efficacy of CAR-T cell therapy (140–142). In this regard, CAR-T cells engineered to express heparanase (HPSE), which degrades heparan sulfate proteoglycans, better infiltrated tumors and had increased antitumor activity in mouse models (23). Since matrix metalloproteinases (MMPs), mainly produced by macrophages, also regulate synthesis and degradation of most of the ECM components, an alternative strategy is to leverage the capacity of macrophages to secrete MMPs and remodel the ECM in order to clear the way for T cells to infiltrate tumors (24). This has been demonstrated in the context of endogenous T lymphocytes, but it could be hypothesized that the use of CAR-macrophages might benefit tumor infiltration of CAR-T cells, although it has not been experimentally tested yet.

### Tumor Vasculature

Aberrant tumor vasculature is required for tumor survival, progression, and metastasis, but also provides a physical barrier for T cell extravasation and infiltration into tumors (144). CAR-T cells capable of destroying tumor vasculature have been developed targeting molecules such as VEGFR-2 (18), VEGFR1 (19), PSMA (20), TEM8 (21), or the fibronectin splice variant EIIIB (22). All of these target antigens are also expressed by a range of tumor cell types, and some of them by immunosuppressive cell populations such as regulatory T cells (T_regs_) and myeloid-derived suppressor cells (MDSCs, i.e., VEGFR2) (145, 146) or by the ECM (i.e., EIIIB), which may improve the outcome of the therapy in patients. Unfortunately, a clinical trial on metastatic cancer patient treated with VEGFR-2 CAR-T cells was terminated due to lack of objective responses (NCT01218867).

## Fibroblasts

CAFs can contribute to up to 90% of the solid tumor mass in carcinomas (147) and represent a complex barrier to entry and activity of endogenous and adoptively transferred immune cells.

CAFs signal in a paracrine fashion with tumor cells and other components of the TME. Tumor promoting CAFs secrete factors, including VEGFs, that induce angiogenesis to improve oxygen and nutrient availability in the tumor. CAFs can also directly provide cancer cells with nutrients, growth factors and immunosuppressive cytokines such as transforming growth factor beta (TGF-β), epidermal growth factor (EGF), platelet-derived growth factor (PDGF), and fibroblast growth factor 2 (FGF2), and serve as a physical barrier to T cell infiltration (148, 149). CAFs heavily contribute to the survival, proliferation, metastasis initiation and, even, de-differentiation of tumor cells into more stem cell-like phenotype (150, 151).

Given their powerful and diverse protumoral effects, an attractive therapeutic approach could be generating CAR-T cells that target CAFs. In addition to eliminating their multiple negative effects, an advantage to targeting fibroblasts would be that they are more genetically stable than tumor cells, so they are less likely to lose antigen expression via immunoediting. Moreover, since mesenchymal tumoral stromal cells are present in almost all human adenocarcinomas, therapies against CAFs could potentially be used for multiple types of tumors (152).

In the setting of solid tumors, different subtypes of CAFs have been proposed to have disparate effects on tumor establishment, growth and progression, as well as in metastatic capacity (25, 153). Therefore, when choosing a CAR-targeted protein, it is important to consider which fibroblast cell subpopulation is going to be depleted (154). With this thought in mind, fibroblast activation protein (FAP) has been proposed as a potentially good target. FAP is a surface peptidase that also has gelatinase activity and is widely expressed in a subset of protumoral fibroblasts in many cancer types (155–157). FAP expression in pancreatic cancer (158, 159) and non-small cell lung cancer (160) is associated with worse clinical outcome. Depletion of FAP+ cells using genetic depletion strategies appeared to enhance T cell mediated antitumor activity in preclinical models of melanoma and pancreatic ductal adenocarcinoma (161–163). Antibodies against FAP have confirmed the suitability of FAP as a target by demonstrating efficient tumor stroma targeting capabilities in clinical trials (157). However, no therapeutic responses were observed, prompting the development of alternative strategies such as FAP antibody conjugates including immunostimulatory antibodies (164) and immunocytokines (165). One of those, an anti-FAP-IL-2v fusion protein, is currently being tested in clinical trials (NCT02627274, NCT03386721) (166). Alternatively, CAR-T cell therapy targeting FAP might be a more potent and efficacious strategy.

### CAR-T Cells Targeting Fibroblasts: A Potential Double-Edged Sword

A number of groups have generated CAR-T cells targeted to mouse FAP and tested their ability to inhibit tumor growth. To date, eight studies have demonstrated antitumor activity of FAP-targeting CAR-T cells in several preclinical models including mesothelioma, lung, mammary, colon, pancreatic cancers (25–32), with a key measure of these studies being the potential for toxicity.

A key concern of targeting FAP is that, while it is highly expressed by CAFs and in wound healing, it is also expressed at low levels in healthy tissues including muscle, adipose tissue, bone marrow mesenchymal stem cells (BMMSCs), skin, and pancreas (167, 168). Complete ablation of FAP-expressing cells in mice using genetic approaches resulted in body weight loss, anemia, bone marrow hypoplasia and pancreatic toxicity (167). With these toxicities in mind, it is of interest to review the studies in which CAR-T cells targeting mouse FAP were tested, however, it is important to recognize that each study used a different single-chain fragment variable (scFv) antibody targeting FAP, different cytoplasmic domains, and different types of T cells (murine vs. human T cells).

Tran and colleagues observed minimal antitumor effect using a CAR with the FAP-5-scFv coupled with mouse CD28, 4-1BB, and CD3ζ intracellular signaling domains, but did observe severe toxicity indicated by significant cachexia and anemia (30). In contrast, Kakarla et al. showed that a FAP-CAR, using the MO35-scFv with human CD28 and CD3 derived domains, controlled tumor burden in a systemic lung carcinoma model without toxicity observed 2 days after T cell injection (27). However, this time point may be too early to see the negative effects exerted by the T cells.

The group at the University of Pennsylvania developed a FAP-CAR containing a scFv from the 73.3 anti-mouse FAP antibody and the human 41BB and CD3ζ intracellular domains (25, 28, 31, 32). These CAR-T cells slowed tumor growth in an immune-response dependent and independent manner in several tumor models in mice. Despite 73.3-FAP-CAR initial efficacy, CAR-T cells isolated from xenograft tumors became hypofunctional (28). Function was augmented by either using mouse T cells from mice lacking the inhibitory enzyme diacylgycerol kinase zeta (DGKZ) (32) or human T cells using the 73.3-CAR linked to the DAP12 signaling domain from natural killer (NK) cells (FAP-KIR CAR) (31). There was a link between enhanced CAR activity and toxicity: while no major toxicities were observed using the “basal” 73.3-FAP CAR-T cells, treatment with the more active DGKZ CAR-T cells resulted in a lymphocytic infiltrate observed in the pancreas (32). Likewise, treatment with the highly active FAP-KIR-CAR resulted in anemia, body weight loss and bone marrow hypoplasia (31). The “basal” 73.3-FAP-CAR targets cells with high FAP densities, like CAFs, while sparing low FAP expressing cells, which may provide a therapeutic window to obtain efficacy in the absence of toxicity. Unfortunately, the 73.3–FAP-CAR is mouse specific and cannot be used in the clinical setting.

There has been one reported clinical trial in which FAP CAR-T cells have been locally injected into the pleural effusion of mesothelioma patients (NCT01722149) (33). The authors reported the route of administration and the therapy to be safe in one patient (34) and, another patient showed stable disease for 1 year (26). Unfortunately, at the time of closure of the clinical trial in mid-2019 only 4 patients had been recruited.

In summary, FAP targeted CAR-T cells have clearly shown some antitumor activity in preclinical models, but they have also demonstrated the potential for toxicity. There does appear to be a viable therapeutic window, however. For this reason, it is likely that the role of FAP CAR-T cells will be in combination therapies. Combining FAP CAR-T cells with tumor-targeted CAR-T cells or with vaccines can result in additive or even synergistic effects (27, 32). Other target proteins like CD10 and GPR77 which identify a newly described CAF subpopulation with protumorigenic functions (169) provide alternative option for CAR development.

## Tumor-Infiltrating Immune Cells as Barriers to Effective Car-T Cell Therapy

**Figure 2:**
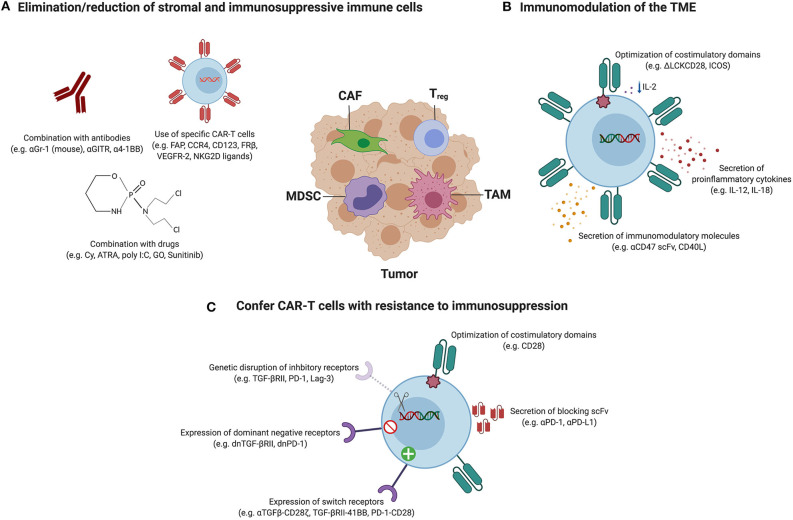
Therapeutic strategies to overcome the immunosuppressive TME. Tumors are infiltrated with stromal cells, such as cancer-associated fibroblasts (CAFs), and immune cells, including regulatory T cells (T_regs_), tumor-associated macrophages (TAMs) and myeloid-derived suppressor cells (MDSCs) which support cancer progression and promote immunosuppression. Therapeutic strategies utilized to hit these components to enhance the efficacy of CAR-T cell therapy can be categorized in three classes, and the most relevant examples are represented in this figure. **(A)** *Elimination or reduction of stromal and immunosuppressive immune cells:* the combination of CAR-T cells with agents such as antibodies or drugs has resulted in decreased frequencies of T_regs_ and/or MDSCs. Alternatively, CARs have been designed to target antigens expressed on CAFs, T_regs_, TAMs and MDSCs to directly deplete them. **(B)** *Immunomodulation of the TME:* this group of strategies aims at manipulating the TME to create a favorable environment that allows a better performance of the CAR-T cells. Some examples include the modulation of the tumor cytokine milieu by the expression of proinflammatory cytokines by CAR-T cells or by the optimization of costimulatory signaling domains in order to reduce IL-2 secretion and therefore impair T_reg_ expansion and tumor infiltration. Immunomodulatory molecules that are able to polarize M2 TAMs into an antitumor M1 phenotype can also be expressed from CAR-T cells. **(C)** *Confer CAR-T cells with intrinsic resistance to immunosuppression:* CAR-T cells can be modified to be resistant to immunosuppression by endowing them with dominant-negative receptors (to disrupt signaling) or chimeric switch receptors (to convert negative signaling into positive), or by abrogating the expression of inhibitory receptors using genome-editing tools. Alternatively, antibodies blocking inhibitory receptors or ligands can be secreted by CAR-T cells. Also, it has been reported that the incorporation of particular costimulatory domains or the expression of some proinflammatory cytokines by CAR-T cells confer intrinsic resistance to T_reg_-mediated immunosuppression.

Solid tumors are highly infiltrated with immune cells such as T_regs_, tumor-associated macrophages (TAMs) or MDSCs that contribute to the establishment of a hostile and immunosuppressive TME capable of limiting the efficacy of CAR-T cell therapy. In this section, we review the obstacles imposed by each of these cell populations and the different strategies that have been utilized in order for CAR-T cells to be efficacious in such context, as illustrated in Figure 2. These include strategies to directly target and deplete the immunosuppressive immune cell populations as well as indirect approaches consisting of genetically engineering the CAR-T cells to endow them with transgenes capable of modulating the TME or to confer them with resistance to immunosuppression.

### Regulatory T Cells

T_regs_ are a subset of T cells (phenotypically defined as CD4^+^CD25^+^FoxP3^+^) which play a crucial role in maintaining immune tolerance to self-antigens but can also suppress antitumor immunity (170). Cancer patients have increased numbers of T_regs_ in peripheral blood (171–173), and their presence in tumors is associated with poor prognosis in a variety of cancers (174–178).

T_regs_ suppress antigen-specific CD8^+^ T cell cytotoxicity by using different mechanisms: competitive consumption of IL-2; secretion of immunosuppressive cytokines such as IL-10 or TGF-β; CTLA-4-mediated suppression of antigen presenting cells (APCs); prevention of optimal T cell activation; or lysis of effector cells through the action of granzyme and/or perforin (170).

In the TME, T_regs_ play an inhibitory role in the antitumor efficacy of adoptively transferred tumor-targeted effector T cells (179). The frequency of T_regs_ in the blood of responder patients was lower than in samples obtained from non-responders in a combined analysis of multiple trials of adoptively transferred TILs (180). In a first-in-human study of an epidermal growth factor receptor variant III (EGFRvIII)-specific CAR in glioblastoma, the analysis of tumor specimens from patients who had post-treatment surgery revealed an increased influx of immunosuppressive T_reg_ cells, which might have limited the antitumor effect of the CAR-T cell therapy (181). In addition, the importance of the effector to regulatory T cell balance in predicting responses to immunotherapy treatments has been highlighted (182).

A first and obvious way to address this limitation is to specifically eliminate T_regs_. The combination of CAR-T cells with antibodies targeting GITR or 4-1BB (whose expression has been reported to be specific for tumor T_regs_) has been explored in mice, resulting in decreased T_reg_ frequencies and enhanced antitumor efficacy (40–43). The idea of directly depleting T_regs_ with a CAR has also been proposed by targeting the C-C chemokine receptor 4 (CCR4), which is expressed on T cell malignancies but also in T_regs_ (44).

Many clinical trials of CAR-T cells have failed to provide significant clinical benefit in the absence of prior lymphodepleting preconditioning (183–188). This lack of success may be explained, at least in part, by the fact that such preparative treatments are known to eradicate T_regs_, which otherwise might suppress infused T cells (189). Illustrating this, the efficacy of CD19 CAR-T cells in a mouse model of lymphoma was completely abolished when T_reg_ cells were previously injected and restored by preparative treatment with cyclophosphamide (45). Unfortunately, preconditioning regimens carry with them toxicities, which in some cases create a need for alternative strategies. In this line, several studies have demonstrated that IL-12 can help to overcome T_reg_-mediated immunosuppression and, therefore, the need for prior preconditioning. In mice, CAR-T cells engineered to constitutively produce IL-12 acquire intrinsic resistance to T_regs_ and are more efficacious in the absence of preconditioning (78, 79). Considering the potential clinical toxicity of constitutive IL-12 expression, safer approaches might involve the use of inducible systems to drive IL-12 production upon antigen recognition or the incorporation of elimination genes (80, 81). Constitutive IL-12-secreting mucin-16 ectodomain (MUC-16^ecto^)-specific CAR-T cells, which also express a truncated form of the human epidermal growth factor receptor (EGFRt) as a safety system, are currently being tested in an ovarian cancer phase I clinical trial (NCT02498912) (82, 83). CAR-T cells expressing alternative cytokines with safer clinical profiles such as IL-18 have also been tested in preclinical models with a similar impact on reducing tumor-infiltrating T_reg_ numbers and improving antitumor activity (84).

A second, indirect approach to overcome CAR-T cell suppression by T_regs_ is to restrain their proliferation and survival by modulating the cytokines in the TME, specifically IL-2. IL-2 sustains the survival and function of both regulatory and effector T cells (190). In fact, IL-2 is often used to improve persistence of adoptively transferred T cells, albeit its administration leads to the expansion of T_regs_ in cancer patients (191). CAR-T cells release high levels of IL-2 upon antigen engagement, becoming a main source of this cytokine. It could be expected, then, that the use of CAR-T cells with reduced levels of secreted IL-2 would improve the antitumor efficacy of these engineered T cells as IL-2 would be no longer available to sustain T_reg_ persistence. Cytokine levels can be modulated by selecting an appropriate co-stimulatory endodomain such as ICOS, which has been reported to generate CAR-T cells with increased IL-17 production and reduced secretion of IL-2 (46). Alternatively, more conventional co-stimulatory domains such as CD28 can be mutated for the same purpose. It is known that IL-2 secretion is initiated by CD28-mediated LCK recruitment and phosphorylation, therefore, the mutation of the LCK binding domain abolishes IL-2 secretion by CAR-T cells (52). This modification improved antitumor efficacy of CAR-T cells in the presence of previously inoculated T_reg_ cells, which persisted less, compared to mice treated with CAR-T cells containing the wild type CD28 endodomain (52).

In apparent contradiction with this study, several groups report that the incorporation of a CD28 co-stimulatory domain in different CAR platforms provides increased resistance to T_reg_-mediated immunosuppression, and more specifically, to TGF-β-mediated suppression of T cell proliferation (47–50). Conversely, while sustaining T_reg_ survival and function, IL-2 induced by CD28 activation of LCK and autocrine signaling through IL-2 receptor on tumor-specific effector T cells appears to be crucial to counteract the inhibitory effects of TGF-β (48, 49). In fact, the deletion of the LCK binding domain in CD28 reverted resistance to TGF-β-mediated suppression (49). One strategy proposed to compensate for the detrimental effect of LCK mutation while maintaining the benefits of abrogating IL-2 secretion is the addition of a 4-1BB co-stimulatory signaling domain (51). A different approach is the use of alternative cytokines to replace the CD28-induced IL-2 autocrine loop. For instance, CAR-T cells can be engineered to express IL-7Rα so that IL-7 can support their function (53). In a more sophisticated approach, CAR-T cells with a disrupted IL-2 axis can be engineered to release transgenic IL-7 and to co-express an IL-7Rα/IL-2β hybrid receptor to provide cell-intrinsic IL-2 signaling through IL-7 (49). Alternatively, cytokine stimulation can be provided by IL-15 through the expression of a tethered membrane-bound IL-15, which has been shown to favor the persistence and survival of CAR-T cells with a clinically desirable immature state of differentiation (54). This interesting approach avoids undesirable effects of soluble IL-15 coadministration or constitutive secretion by CAR-T cells such as o toxicity (192) or promotion of T_regs_ (193). In addition, there is great excitement on the use of engineered IL-2 mutants designed to preferentially signal into effector T cells but not T_regs_, although this strategy has not yet been tested in the context of CAR-T cells (194–196).

Besides suppressing T cell proliferation, TGF-β induces a T_reg_-like phenotype on CAR-T cells (77). Therefore, conferring CAR-T cells with intrinsic resistance to TGF-β represents an opportunity for improvement. TGF-β signaling in CAR-T cells can be abrogated by knocking out TGF-βRII through CRISPR/Cas9 technology (77). In the same line, CAR-T cells can be endowed with a TGF-β dominant-negative receptor (dnTGF-βRII). A first-in-human trial in patients with refractory castration-resistant metastatic prostate cancer has been initiated with a prostate-specific membrane antigen (PSMA)-specific CAR incorporating this receptor (NCT04227275) (74). Alternatively, switch receptors can be created by fusing the extracellular part of the TGF-βRII to the endodomain of 4-1BB or by linking a TGF-β-specific scFv to the CD28-CD3ζ intracellular signaling domains, rendering CAR-T cells capable of converting the immunosuppressive signal from soluble TGF-β into an immunostimulatory one (75, 76).

### Tumor-Associated Macrophages

TAMs are the most abundant immune cells infiltrating human cancers and their accumulation in tumors correlates with poor prognosis in a broad range of tumor types (197, 198). TAMs can sustain cancer progression by secreting growth factors which stimulate tumor cell proliferation, proteolytic enzymes that promote matrix remodeling and facilitate metastasis, proangiogenic factors which support angiogenesis, or reactive oxygen species (ROS) and nitric oxide (NO) that induce genetic instability on tumor cells (199). Furthermore, TAMs can suppress T cell-mediated antitumor immunity by releasing IL-10 and TGF-β, amino acid-depleting enzymes such as arginase 1 or indoleamine 2,3-dioxygenase (IDO) which cause metabolic starvation on T cells or prostaglandins with immunosuppressive effects, or by expressing immune checkpoint ligands like PD-L1, PD-L2, B7-H4, or VISTA. Moreover, TAMs can promote the recruitment and immunosuppressive activity of T_regs_ (199). TAMs can also prevent T cell-mediated antitumor immune responses by physically creating long-lasting interactions with CD8^+^ T cells, thus excluding them from tumors (200).

There is overt preclinical evidence of that TAMs can mediate resistance to immunotherapy, including adoptive cell transfer therapy. For instance, the depletion of TAMs through the administration of a CSF-1R inhibitor improved the efficacy of adoptively transferred tumor-specific T cells in syngeneic mouse models of melanoma (200). Superior antitumor activity of the combined treatment correlated with a decrease in the number of intratumoral macrophages, which subsequently facilitated an increase in expansion, intratumoral accumulation and functionality of the adoptively transferred T cells (201).

In the field of CAR-T cell therapy, the infusion of GD2-specific CAR-T cells in neuroblastoma patients provoked a striking expansion of circulating macrophages with immunosuppressive phenotype suggesting a role of macrophages limiting the antitumor efficacy (88).

Despite their overall tumor-promoting functions, certain subpopulations of TAMs can sustain antitumor activities including phagocytosis, antigen-presenting, or the release of proinflammatory cytokines such as TNF-α and IL-12. Indeed, in certain contexts, macrophages have been proven crucial for the development of effective immunotherapy (202–204). Several strategies have been proposed to either reprogram immunosuppressive “M2-like” TAMs into an antitumor “M1-like” phenotype which could cooperate with CAR-T cells to induce tumor regression, or to directly deplete TAMs to facilitate productive antitumor immunity.

One strategy of TAM reeducation consists in making them more phagocytic. CD47 is expressed on tumor cells and interacts with SIRPα expressed on macrophages to deliver a “don't eat me” signal. CAR-T cells can be engineered to express CD47-blocking antibodies in order to prevent that interaction, thus stimulating phagocytosis of tumor cells and improving engagement of the innate immune system (58, 59). A clever approach to hijack the phagocytic capacities of TAMs and redirect them toward tumor-associated antigens is to engineer macrophages themselves to express a CAR. Interestingly, macrophage transduction with chimeric adenoviral vectors promoted a gene expression change toward a proinflammatory M1 phenotype, which subsequently converted bystander M2 TAMs into an M1 phenotype and boosted endogenous antitumor T cell responses (60).

TAMs can also be manipulated to become more functionally activated. CD40 is expressed in antigen presenting cells (APCs) including dendritic cells (DCs), B cells, monocytes and macrophages. Interaction of CD40 with its ligand, CD40L, is known to induce activation and IL-12 secretion by APCs. Preliminary studies using a bispecific antibody to mediate the interaction between a c-myc tag on CAR-T cells and CD40 on APCs demonstrated enhanced CAR-T cell function (61). Constitutive expression of CD40L by CAR-T cells improved their therapeutic efficacy in part through the induction of maturation and IL-12 secretion by monocyte-derived DCs and macrophages (62, 63). By means of a different pathway, the administration of the multikinase inhibitor sorafenib in combination with CAR-T cells also induced an increase in IL-12 production by TAMs which contributed to antitumor activity (64).

Not surprisingly, cytokines secreted by CAR-T cells upon antigen encounter can alter the TME and convert TAMs from immunosuppressive to immunostimulatory. For instance, secretion of GM-CSF and IFN-γ by CAR-T cells upon antigen engagement has been shown to elicit a recruitment of myeloid cells to the TME and to activate newly recruited as well as re-educate resident suppressive TAMs thus potentiating their IL-12 production, capacity of antigen presentation, and tumoricidal activity (205). Armoring CAR-T cells with additional cytokines can improve their capacity to modulate the TME. In mice, inducible IL-12 secretion by CAR-T cells resulted in the recruitment of activated TNF-α-producing macrophages which directly contributed to tumor elimination in a TNF-α-dependent manner (206). In addition, IL-12 secretion by CAR-T cells indirectly mediated the depletion of TAMs as a result of Fas engagement on TAMs by FasL on CAR-T cells and altered the phenotype of remaining TAMs toward a proinflammatory one (83). IL-18-secreting CAR-T cells also led to a reduction in “M2-like” macrophages in tumors as well as T_regs_ (84).

A different strategy to overcome immunosuppression in the TME is to develop CARs that target antigens expressed by TAMs to directly eliminate them. CAR-T cells targeting the antigen CD123, with shared expression in malignant cells and TAMs, have been proposed for the treatment of Hodgkin lymphoma which contains a highly immunosuppressive TME (55). Alternatively, rather than hitting all macrophages by using a pan-macrophage target, it would be desirable to design CARs that are able selectively deplete TAMs with protumor “M2-like” properties while sparing other TAM populations with antitumor “M1-like” functions. CAR-T cells targeting folate receptor β (FRβ), which is expressed only in the immunosuppressive TAM population, have been developed for that aim (56). Similarly, CAR-T cell targeting B7-H4, a molecule expressed by cancer cells and TAMs, mediated antitumor responses in a preclinical ovarian cancer model, but was also toxic due to possible targeting of tissue resident macrophages (57).

Finally, CAR-T cells can also be combined with agents that protect them from TAM-related immunosuppressive pathways, such as that mediated by IDO. IDO is produced by tumor cells and TAMs and mediates the metabolism of tryptophan into immunosuppressive metabolites that can suppress CAR-T cell function. The use of IDO inhibitors or preconditioning with fludarabine, which can inhibit IDO expression, are strategies that can be used to improve the activity of CAR-T cells in immunosuppressive microenvironments (85).

### Myeloid-Derived Suppressor Cells

MDSCs are a highly diverse population of immature myeloid cells which include two major subsets: the mononuclear MDSCs (M-MDSCs), which are morphologically and phenotypically similar to monocytes and can differentiate into TAMs, and the polymorphonuclear MDSC (PMN-MDSCs), which resemble neutrophils and are precursors of tumor-associated neutrophils (TANs), as well as a small group of myeloid progenitors (207). MDSCs play a role in supporting tumor progression, and according to a meta-analysis of the literature, their accumulation is associated with poor clinical outcome in cancer patients (208). The hallmark feature of MDSCs is their strong capacity to inhibit immune responses, with T cells being the main targets of these effects. Mechanisms implicated in MDSC-induced immunosuppression are common to those reported for TAMs, including production of NO and ROS, elimination of key nutrition factors needed for T cell proliferation such as arginine, cysteine, or tryptophan, production of IL-10 and TGF-β, and induction of T_regs_ (209). MDSCs have also been implicated in limiting the effects of CAR-T cell therapy. In a clinical trial of third generation CD19 CAR-T cell therapy, low levels of M-MDSCs was associated with response in patients with lymphoma and leukemia (210).

The detrimental effect of MDSCs on CAR-T cell proliferation and cytolytic function has been demonstrated by using CARs targeting a number of different antigens (65–67). As a proof of concept, depletion of MDSCs with anti-Gr-1 antibody resulted in improved antitumor efficacy of CAR-T cells in mouse models (40, 65, 66). Unfortunately, the lack of a suitable marker for human MDSCs prevents their targeting by using a single antibody. It has been demonstrated that GM-CSF and STAT3 signaling through GM-CSF and/or IL-6 can drive the expansion of MDSCs and support PD-L1 expression by these cells, promoting suppression of CAR-T cells through the PD-1/PD-L1 axis. Therefore, GM-CSF neutralization, STAT3 inhibition or PD-L1 blockade might represent alternative targets to limit the impact of MDSCs in humans (65, 68). The combination of CAR-T cells with compounds such as polyinosinic-polycytidylic acid (poly I:C), all-trans retinoic acid (ATRA), gemtuzumab ozogamicin (GO) or sunitinib also resulted in improved antitumor efficacy attributed to a reduction in the content and suppressive function of MDSCs (66, 67, 69, 70).

Interestingly, some studies combining CAR-T cell therapy with anti-PD-1 or anti-4-1BB antibodies have reported a decrease in the percentage of MDSCs in the TME, correlating with improved antitumor effects (43, 71). However, mechanisms underlying MDSC depletion mediated by immune checkpoint blockade are not fully understood.

A more direct approach of depleting MDSCs by using CAR-T cell therapy is to target antigens expressed on their surface. For instance, CAR-T cells targeting tumor vasculature through VEGFR-2 were able to reduce the frequency of MDSCs in the TME, which also expressed VEGFR-2 (72). Parihar and colleagues engineered NK cells to express a chimeric activating receptor comprised of the extracellular domain of NKG2D receptor fused to the T cell signaling domain CD3ζ (73). Engineered NK cells achieved efficient depletion of MDSCs, which express NKG2D ligands, and increased the recruitment and tumor infiltration of tumor-specific CAR-T cells when given in combination.

Neutrophils can also be immunosuppressive in the context of cancer, and their presence in tumors has been associated with poor outcome (211). In a CAR-T cell therapy trial targeting CEA, increased neutrophil to lymphocyte ratios correlated with poor responses in colon cancer patients with liver metastasis (212). Like TAMs, tumor-associated neutrophils (TANs) can be generally classified into antitumorigenic “N1” or protumorigenic “N2” phenotypes (213). Although strategies to target “N2” TANs have not been reported yet in the context of CAR-T cell therapy, some of the above-mentioned strategies could be used to counteract immunosuppressive pathways common with T_regs_, TAMs, or MDSCs.

## Inhibitory Receptors and Their Ligands

Tumor cells, tumor-infiltrating immune cells and tumor-derived exosomes frequently express an array of ligands that bind to inhibitory receptors on T cells to suppress antitumor immunity. Blocking these interactions with therapeutic antibodies, known as immune checkpoint inhibitors, releases the brakes from suppressed T cells, allowing them to recover their antitumor activity. This therapeutic approach can mediate long-term responses, especially in a subset of tumors that are infiltrated with neoantigen-specific T cells. Therapeutic antibodies targeting the inhibitory receptors CTLA-4 and PD-1 or the PD-1 ligand PD-L1 have been approved for clinical use in patients with different solid cancer types (9). Checkpoint blockade has revolutionized cancer treatment, highlighting the tremendous power of T cells in controlling solid tumors.

Among the different immune checkpoints, the PD-1/PD-L1 axis has gained increasing attention. PD-1 is expressed in the surface of activated or dysfunctional T cells, while PD-L1 is frequently expressed in the surface of tumor cells and immune cells, and can also be found in extracellular forms (214, 215). PD-L1 upregulation is mainly associated with IFN-γ release in response to T cell activation (216); however more recent findings suggest that multiple cytokines found in the TME (including IL-10, IL-1α, IL-27, and IL-32γ) can induce PD-L1 expression (217). Of note, some cancer cells can constitutively express the PD-L1 gene due to hypomethylation of its promoter, while TAMs have been reported to also express PD-L1 naturally or via trogocytosis from tumor cells (218). Expression of PD-L1 in the tumor restrain tumor infiltrating lymphocytes from full and persistent activation. Moreover, PD-L1 expression in the stroma can prevent T cells from infiltrating the tumor, excluding them to the margin of the tumor (219). Blocking the PD-1-PD-L1 interaction can promote T cell proliferation and infiltration into the tumor, and results in durable antitumor responses (219).

The success of checkpoint immune therapies targeting CTLA-4 or the PD-1/PD-L1 axis has prompted intense investigation into new inhibitory receptors, including TIM-3, LAG-3, and TIGIT. A new wave of therapeutic agents targeting these receptors are being investigated in clinical trials, with encouraging initial results (220). However, little is known about the biology of these receptors and the interactions with their ligands. TIM-3 ligands include the cell surface ligands Ceacam-1 and Phosphatidyl serine-PTdSer (221) and the soluble factors, Galectin-9 (222) and HMGB1, that are released to the TME. LAG-3 also interacts with various ligands in the TME, including MHC class II expressed in APC and tumor cells; Galectin-3 (223) and LSECtin, expressed on tumor-associated stromal cells and tumor cells; and FGL-1, a soluble factor produced in some tumors (224). TIGIT interacts with the ligands CD112 and CD155, which are expressed on APCs and tumor cells. Expression of these ligands in tumors is associated with tumor progression and inhibition of antitumor T cell responses (224–227).

### Releasing the Breaks on CAR-T Cells

A promising strategy to increase the antitumor efficacy of CAR-T cells is to prevent or revert T cell dysfunction driven by engagement of inhibitory receptors with their ligands in the tumor. Upon antigen recognition, CAR-T cells up-regulate different inhibitory receptors, similarly to endogenous tumor-specific T cells. CAR-T cells isolated from xenograft tumors typically express high PD-1 levels, with a fraction of these cells co-expressing TIM-3 and LAG-3 (86, 228). Overexpression of PD-L1 by tumor cells has been shown to inhibit CAR-T cell function, while combining CAR-T cell therapy with antibodies that block the PD1/PD-L1 interaction has proved to increase the antitumor effects of each therapy alone (71, 86, 87). One study using syngeneic mouse models showed therapeutic responses when combining CAR-T cells with PD1-blocking antibodies, which was correlated with a decrease in MDSCs (71). Several ongoing clinical trials are testing the combination of CAR-T cells with anti-PD-1/PD-L1 blocking antibodies in patients with hematologic malignancies or solid tumor (NCT02414269, NCT01822652, NCT03980288, NCT03726515), with some preliminary results with small groups of patients showing safety and encouraging efficacy results (88–90).

Novel alternative approaches to target the PD-1/PD-L1 axis include the genetic modification of CAR-T cells to release a PD-1- or PD-L1-blocking scFv in the tumor (92, 93), to express PD-1 dominant negative receptors (86), or chimeric switch receptors (94). These strategies may avoid the toxicities associated with systemic delivery of checkpoint inhibitors and bypass the requirement for repeated antibody administration. Expression of chimeric switch receptors has the advantage of converting an inhibitory signal (PD-1) into a costimulatory signal (i.e., CD28) (94). Compared to PD-1 chimeric receptors, the delivery of PD-1 or PD-L1 blocking antibodies (by combination therapy or genetic modification) offers the possibility to re-invigorate endogenous tumor-specific T cells (92), which may be required to achieve complete responses in solid tumors. In this line, combination of CAR-T cells with oncolytic viruses releasing an anti-PD-L1 mini-body locally in the tumor resulted in enhanced therapeutic effects (91). Oncolytic viruses provide a danger signal able to diminish tumor immunosuppression while inducing tumor debulking, and may be ideal partners to combine with CAR-T cells and immune checkpoint inhibitors (229).

Another strategy to counteract tumor-induced T cell inhibition is to disrupt T cell inhibitory receptors by genome editing. Several studies have demonstrated that PD-1 gene editing, using TALEN or the CRISPR/Cas9 system, can augment T cell-mediated killing *in vitro* and enhance clearance of PD-L1^+^ tumors *in vivo* (95–97). However, reported *in vivo* results testing this strategy seem to be contradictory and conflicting. Recent studies suggest that PD-1 ablation or knockdown can accelerate T cell exhaustion, prevent memory formation and reduce long-term antitumor efficacy (230, 231). Enhanced antitumor effects with PD-1 knockout (KO) CAR-T cells are usually observed in animal experiments using tumor cell lines genetically modified to express constitutive and uniform levels of PD-L1. So, it is possible that PD-1 disruption is only beneficial in tumors with high PD-L1 tumor densities. Different clinical trials are actively testing PD-1 KO engineered T cells for the treatment of solid tumors (NCT03747965, NCT03525782, NCT03706326, NCT03399448). A first-in-human phase 1 clinical trial has recently published the safety and feasibility of deleting three genes (TRAC, TRBC, and PDCD1, the gene encoding PD-1) using CRISPR-Cas9 in cancer-specific T cells for the treatment of patients with refractory cancer (98). Initial results in three patients demonstrated engraftment of PD-1–deficient T cells with no evidence of autoimmunity or T cell genotoxicity. Surprisingly, it was found that, in one patient, the percentage of tumor-specific T cells with mutations in the PD-1 locus decreased from 25% in the infusion product to 5% 4 months post-infusion. While further investigations are required to interpret these results, loss of PD-1 edited T cells would be consistent with mouse studies highlighting the role of PD-1 in preserving T cells from overstimulation and terminal differentiation. In this same line, initial reports have established the feasibly of knocking out other inhibitory receptors, such as CTLA-4 or LAG-3, but it remains unclear as to whether these modifications result in enhanced CAR-T cell activity (99, 100). A better understanding on the mechanisms by which inhibitory receptor negatively regulate T cell function together with preclinical models that better recapitulate the TME are required to design the next-generation CAR-T cell therapies.

## Conclusions and Future Directions

Unprecedented durable responses in cancer patients treated with checkpoint blockade antibodies or CAR-T cell therapy is generating considerable optimism. Augmenting the therapeutic outcome of CAR-T cell therapy in the context of solid tumors represents the next big challenge and opportunity for the field. Clearly, a major obstacle for CAR-T cells in solid tumors is the immunosuppressive TME. There is now an understanding that physical barriers and stromal and immune cells that express and release an array of immunosuppressive molecules limit CAR-T cell persistence and efficacy. In these hostile circumstances, strategies aimed at remodeling the tumor microenvironment or conferring intrinsic CAR-T cell resistance to immunosuppression may be more promising than targeting only one specific pathway. The cellular component of TME is characterized by considerable diversity and a high degree of plasticity (232, 233). Several strategies directed to regulating this plasticity and reversing immunosuppression are being explored. Armored CAR-T cells expressing proinflammatory cytokines or combination of CAR-T cells with oncolytic viruses could serve this purpose (234). Gene ablation technology will allow CAR-T cells to avoid immunosuppresssive signals in the TME. By a different approach, direct elimination of stroma or immune suppressive cells could revert immunosuppression, tackling different pathways simultaneously. Ongoing efforts seek to develop a new generation of CAR-T cell therapies targeting fibroblasts, T_regs_, M2 macrophages or MDSCs.

Other factors such as the effect of gut microbiota on response to immune therapies might be also considered. It has been recently reported by many groups that microbiome composition modulates the antitumor response to immune checkpoint inhibitors. This effect is described to be mediated by IL-12 and to correlate with a decrease of T_regs_ and MDSCs in the TME (235). Similar observations have been made in preclinical mouse studies in the context of adoptive cell transfer therapy (236). In the field of CAR-T cell therapy, a preliminary study of microbiota composition in cancer patients prior to CAR-T cells infusion found a correlation between the presence of certain bacterial families and efficacy and toxicity of the therapy (237). This observation warrants future consideration of strategies such as the use of specific antibiotics or fecal microbial transplantation in combination with CAR-T cell therapy (238).

One of the greatest challenges in developing effective and safe CAR-T cells that tackle the TME is the lack of clinically relevant models that reflect the challenges of solid tumors. Currently available preclinical models have been unable to predict the toxicities observed in clinical trials and the lack of antitumor activity, especially in patients with solid tumors. Advanced preclinical models relevant to study the impact of tumor heterogeneity and the role of the TME in CAR-T cell efficacy are required to test the next-generation of CAR-T cells as monotherapy or in combination with other agents. The testing of such CAR-T cell approaches in canines with spontaneous solid cancer represents a promising avenue of investigation (239). Current clinical studies will hopefully reveal information on the safety and efficacy of novel CAR-T cell approaches, including those addressing barriers of the TME. Lessons learned from these early-phase clinical trials will be important to continue to develop novel CAR-T cell therapies for the treatment of solid tumors.

## Author Contributions

SG and AR-G conceptualized, wrote, and edited the manuscript. AP and EN-O wrote and edited the manuscript. AR-G and AP designed the figures. DP edited the manuscript.

## Conflict of Interest

DP and SG are inventors on patents related to CAR-T cell therapy, filed by the University of Pennsylvania and licensed to Novartis. The remaining authors declare that the research was conducted in the absence of any commercial or financial relationships that could be construed as a potential conflict of interest.
